# Thermostable exoshells fold and stabilize recombinant proteins

**DOI:** 10.1038/s41467-017-01585-2

**Published:** 2017-11-13

**Authors:** Siddharth Deshpande, Nihar D. Masurkar, Vallerinteavide Mavelli Girish, Malan Desai, Goutam Chakraborty, Juliana M. Chan, Chester L. Drum

**Affiliations:** 1 Cardiovascular Research Institute, Department of Medicine, Yong Loo Lin School of Medicine, National University of Singapore, 1E Kent Ridge Road, NUHS Tower Block, Level 9, NUHCS, Singapore, 119228 Singapore; 2 Department of Medicine, Yong Loo Lin School of Medicine, National University of Singapore, Singapore, 119228 Singapore; 3 NUS Graduate School for Integrative Sciences and Engineering, National University of Singapore, Singapore, 117456 Singapore; 4 School of Chemical and Biomedical Engineering, Nanyang Technological University, 70 Nanyang Drive, Singapore, 637457 Singapore; 5 Lee Kong Chian School of Medicine, Nanyang Technological University, 59 Nanyang Drive, Singapore, 636921 Singapore; 6 Translational Laboratory in Genetic Medicine, 8A Biomedical Grove, Immunos, Level 5, Singapore, 138648 Singapore; 7 Department of Surgery, Yong Loo Lin School of Medicine, National University of Singapore, Singapore, 119228 Singapore

## Abstract

The expression and stabilization of recombinant proteins is fundamental to basic and applied biology. Here we have engineered a thermostable protein nanoparticle (tES) to improve both expression and stabilization of recombinant proteins using this technology. tES provides steric accommodation and charge complementation to green fluorescent protein (GFPuv), horseradish peroxidase (HRPc), and *Renilla* luciferase (rLuc), improving the yields of functional in vitro folding by ~100-fold. Encapsulated enzymes retain the ability to metabolize small-molecule substrates, presumably via four 4.5-nm pores present in the tES shell. GFPuv exhibits no spectral shifts in fluorescence compared to a nonencapsulated control. Thermolabile proteins internalized by tES are resistant to thermal, organic, chaotropic, and proteolytic denaturation and can be released from the tES assembly with mild pH titration followed by proteolysis.

## Abstract (web summary)

Improving recombinant protein expression and stabilization remains a significant challenge. Here, the authors engineer *Archaeoglobus fulgidus* ferritin as a thermostable exoshell to provide steric accommodation and charge complementation for recombinant proteins, which can improve yields by 100 fold.

## Introduction

The production and stabilization of recombinant protein remains a significant challenge for basic and industrial science. Technologies to improve protein folding have had varied success, ranging from chaperone coexpression to chemically engineered hydrogels^1–3^. Methods to stabilize protein products, including chemical cross-linking, rational mutagenesis, and directed evolution approaches, have also found use in basic and industrial applications^1–4^.

Thermophilic organisms have evolved unique solutions for protein folding and stabilization due to the extreme conditions in which they live. Although engineered thermostable proteins have had major impacts in basic and applied biology^5^, this approach is limited by the observation that many protein functionalities found in higher-order organisms have no homologs within the biology of prokaryotic thermophiles. Thus, general technologies which can impart thermostable qualities, in particular improved folding and stability, to normothermic substrates remain highly desirable.

**Fig. 1:**
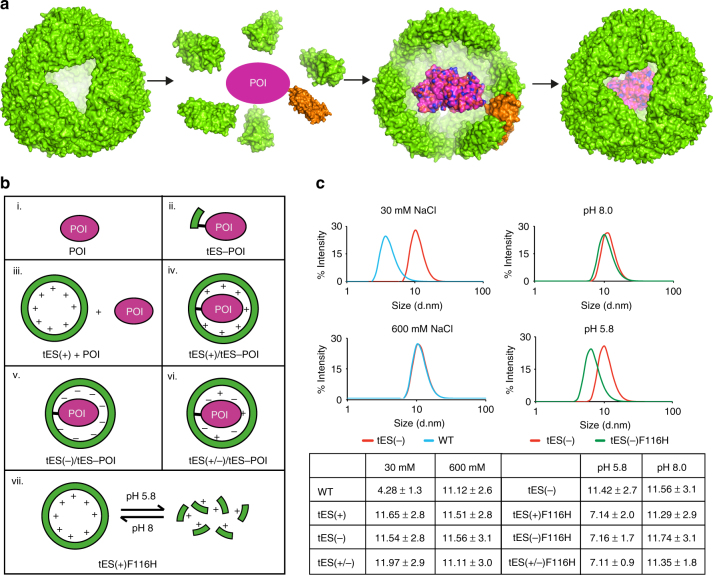
Engineering of six thermostable exoshell variants. **a** Overview of the encapsulation process, as generated using PyMOL v1.8.6.0. **b** Schematic of terminologies. (i) POI, protein of interest; (ii) thermostable exoshell-POI (tES-POI), POI fused to the C-terminus of a tES subunit; (iii) POI expressed in the presence of a tES shell but without encapsulation; (iv–vi) coexpression of tES-POI with tES(+), tES(−), and tES(+/−) shells; and (vii) tES(+)F116H, pH-titratable subunits. **c** Dynamic light scattering of tES variants. Mutation of F116 to H resulted in a pH-titratable assembly and dissociation. The results are expressed as means ± standard deviation (SD) (*n* = 3)

*Archaeoglobus fulgidus* is a hyperthermophilic halophile naturally found in hydrothermal vents and subsurface oil fields^6, 7^. Both eukaryotic and *A. fulgidus* ferritins (AfFtn, PDB accession code: 1SQ3) contain 24 subunits, with each subunit containing a single four-helix-bundle motif. AfFtn is different from typical ferritins in two important ways. First, the unique, tetrahedral (2–3) symmetry of the AfFtn quaternary structure results in four ~ 4.5-nm pores, which connect the internal and external volumes of the shell (Fig. 1a)^8, 9^. Second, while eukaryotic ferritin assemblies are stable in low-salt concentrations^10^, AfFtn disassociates in low-salt (NaCl) concentrations^8^.

To improve recombinant protein expression and product stabilization using a single technology, we have engineered the AfFtn assembly derived from *A. fulgidus*, to create a thermostable exoshell (tES). tES accommodates foreign proteins within an 8-nm aqueous cavity while, at the same time, allowing internalized enzymes to have an access to molecular substrates through four 4.5-nm pores present in the native structure of the shell. We hypothesize that tight steric and electrostatic complementarity to an internalized substrate prevents aggregation during the folding process while stabilizing correctly folded tertiary structures (Fig. 1 and Supplementary Fig. 1). We also demonstrate that tES is protective against a wide range of denaturants, thus imparting thermostable qualities to internalized normothermic proteins.

## Results

### Engineering of tES

AfFtn subunits have unstructured negatively charged C-termini, which protrude into the central cavity of the 24-subunit AfFtn assembly. Together, these C-termini have a molecular mass of ~ 21 kDa, which would likely interfere with the internalized protein folding and function. Therefore, we created a truncation mutant at residue 164 (Q164), to remove unstructured amino acids from the cavity and create the template for “tES.” Surprisingly, the modified AfFtn assembly was stable in all salt concentrations tested (Fig. 1c). A circular dichroism study of tES(+), tES(−), and tES(+/−) shells showed stability of a secondary structure to 80 °C; however, partial denaturation was observed at 90 °C (Supplementary Fig. 2).

Given the proximity between the internal cavity surface and internalized recombinant peptides, we further modified the charge characteristics of the tES aqueous cavity to present either net positive [tES(+)], negative [tES(−)], or neutral [tES(+/−)] charge environments to an internalized protein of interest (POI) (Fig. 1b and Supplementary Fig. 1b). All tES constructs showed a high expression (Supplementary Fig. 3) with an estimated yield of 412, 402, and 237 mg/L (approximately 36, 35.23, and 20.76%, respectively, of the total bacterial protein estimated by densitometry using ImageJ software) for tES(+), tES(+/−), and tES(−), respectively (Supplementary Fig. 3b–d) and the expected values for a diffusional radius and stability (Supplementary Fig. 3e, f).

### Effect of F116H mutation on shell assembly

Because the engineered tES assembly was highly stable in both low- and high-salt concentrations, we further engineered a pH-titratable mechanism to control tES assembly and release. F116 exists along a 3-fold symmetry axis and its substitution to histidine would be expected to create mutual charge repulsion with H104, located on a neighboring subunit, upon protonation (Supplementary Fig. 4a). As expected, tES(+)F116H, tES(−)F116H, and tES(+/−)F116H show a reversible disassociation at pH 5.8 and a stable assembly at pH 8.0 (Fig. 1c and Supplementary Fig. 4b, c).

### Soluble expression of proteins in the presence of tES

To demonstrate the effect of tES on recombinant *Escherichia coli* expression and in vitro folding, we prepared genetic fusions (tES-POI) between a histidine-tagged tES monomer and three divergent POI (Supplementary Fig. 1c). Green fluorescent protein (GFPuv, 27 kDa) was chosen due to its highly variable expression in *E. coli*, prior use in the identification of productive folding environments, and ease of functional measurement^11, 12^. We chose *Renilla* luciferase (rLuc, 36 kDa) due to an absence of previous reports of successful in vitro refolding, its relatively high thermolability, and ease of the functional assay as a bioluminescent reporter enzyme^13^. Finally, we chose a truncated version of the widely used industrial enzyme, horseradish peroxidase (HRPc, 34 kDa), as the highest reported soluble yield for full-length HRP in *E. coli* is approximately 0.5 mg/L culture and for the truncation mutant of a native enzyme, there are no reports of soluble expression in *E. coli* ^14^. HRPc is a more complex POI substrate for in vitro and in vivo folding because it requires an exogenous heme prosthetic group and the formation of four disulfide cross-links for enzyme function^14–17^.

To test the effect of coexpression with tES for the three fusion proteins, we developed a two-plasmid system. The tES was expressed using a high-expression T7 promoter (pRSF) and the tES-POI fusion subunit was expressed using L-arabinose induction from a highly titratable, complementary plasmid (pBAD). Induction was initially optimized with the tES(+)/tES-GFPuv fusion (Supplementary Fig. 1c). To demonstrate tES rescue of GFPuv expression, GFPuv was induced at 0.8 (O.D. 600 nm) due to its extremely low expression in late exponential phase^10^. While leaving isopropyl β-D-1-thiogalactopyranoside (IPTG) concentration constant (0.4 mM), L-arabinose was varied from 0.001 to 0.1%. The maximum functional and soluble expression of tES-GFPuv (Supplementary Fig. 3a) occurred at 0.01%. While the expression of all three POIs separate from tES showed a negligible soluble expression (with the exception of rLuc, which is previously reported to result in soluble expression in *E. coli*)^18, 19^, coexpression of tES-POI with tES resulted in easily visualized bands in the soluble fraction in all three cases (Supplementary Fig. 3b–d). The tES(+) shell resulted in the highest relative concentration of a soluble product, with yields of 79.5, 74, and 57 mg/L of GFPuv, HRPc, and rLuc, respectively (Supplementary Fig. 3b–d), as analyzed by densitometry using ImageJ software. We hypothesize that this may be due to charge complementation between the net-negative surface charge of the POIs and the net-positive charge of the tES(+) internal surface. Assembly of tES(+)/tES-POI was confirmed by pull down of shell components by the histidine-tagged fusion subunit, followed by size-exclusion chromatography (SEC) to confirm tES/tES-POI assembly (Supplementary Fig. 3e, f). The ratios of tES-GFP, tES-HRP, and tES-rLuc to encapsulating tES subunits were in approximate agreement with the expected value of 1:23 (1:32, 1:19, and 1:20, respectively), as estimated by gel densitometry of purified protein fractions.

Using coexpressed tES(+)/tES-POI, we optimized postexpression protocols for improved yield after in vivo assembly within *E. coli*. We focused on HRPc as it requires disulfide formation and a prosthetic group. We confirmed that only by adding the known cofactors heme and calcium, in addition to oxidizing conditions, does maximum functional yield result after harvesting soluble tES(+)/tES-HRPc from *E. coli* lysates (Supplementary Fig. 5a).

### Effect of different tES:tES(+)-GFPuv ratios protein folding

**Fig. 2:**
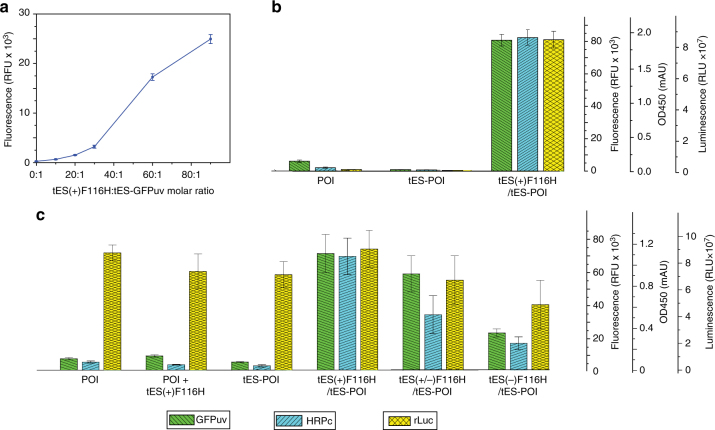
Effects of tES variants on the functional yield of internalized peptides. **a** Effect of different molar ratios of tES(+) subunits to tES-GFPuv on GFPuv activity using in vitro folding. **b** Functional analysis of the in vitro-folded GFPuv, HRPc, and rLuc in the presence and absence of tES by measurement of fluorescence, absorbance, and luminescence, respectively. **c** Analysis of GFPuv, HRPc, and rLuc under a combination of coexpression conditions measured by fluorescence, absorbance, and luminescence, respectively (details in Fig. 1b). All experiments were performed in triplicates. Error bars represent means ± standard deviation (SD)

We tested the ability of tES to aid in vitro folding of proteins. The tES(+)F116H assembly is highly stable at pH 8.0, as evidenced by similar elution profiles on SEC after treatment with 8 M urea or 6 M guanidinium hydrochloride (GuHCl), compared with PBS controls. Likewise, tES(+)F116H can reversibly associate and dissociate with pH titration with no observable precipitates (Supplementary Fig. 4c). We then hypothesized whether tES could functionally encapsulate substrate proteins under conditions that would denature the POI. tES fusion proteins expressed as inclusion bodies in the absence of coexpressed tES (Supplementary Fig. 3b–d). Thus, using tES(+)F116H, we tested the ratios of tES to tES-POI, using a pH shift from 5.8 to 8.0 to induce assembly of the shell. The addition of tES(+)F116H to tES-GFPuv resulted in an ~ 100-fold increase in functional yield, which was maximum at a 90:1 ratio of tES(+)F116H subunits to tES-GFPuv (Fig. 2a and Supplementary Fig. 5b, c). We found that heating the inclusion body suspension to 60 °C in the presence of 6 M GuHCl and 10 μM β-mercaptoethanol was critical for a maximum final yield.

Based on pH titration results (Fig. 2a), we developed a standard protocol with a 60:1 ratio of tES(+)F116H:tES-POI, starting with all components in 6 M GuHCl at pH 5.8. Protein-specific, posttranslationally required agents were added, and the solution was then dialyzed against GuHCl-free buffer at pH 8.0 (Supplementary Fig. 6). Using this protocol, we demonstrate a near-absolute requirement for tES for functional in vitro folding of tES-GFPuv, tES-rLuc, and tES-HRPc. Under the same protocol, very little or no functional yield of GFPuv, HRPc, or rLuc, was observed in the absence of tES (Fig. 2b). A similar pattern of protein activity was seen in *E. coli* lysates with the exception of rLuc, which is known to be expressed as a soluble protein in *E. coli* (Fig. 2c)^18, 19^.

### Iron uptake qualities of tES

The native AfFtn shell is a physiologic iron-storage protein; we investigated whether tES variants also retain iron, either in our purified preparation or when exposed to an in vitro iron-loading protocol^20^. Equimolar amounts (1 µM) of tES(+), tES(−), tES(+/−), and commercially available horse spleen ferritin were compared, and we observed no detectable iron core in purified tES variants (Supplementary Fig. 7a). We then tested iron uptake using equimolar amounts (1 µM) of wild-type AfFtn, tES(+), tES(−), tES(+/−), tES(+)F116H, tES(+)F116H/tES-GFPuv, tES(+)F116H/tES-HRPc, and tES(+)F116H/tES-rLuc. Wild-type AfFtn showed the highest in vitro iron uptake, while empty tES shells had appreciable but lower iron accumulation, likely due to amino acid substitutions near the ferroxidase center (E128 and E131)^6^. A comparison of tES(+) and tES(+)F116H iron uptake was qualitatively similar, indicating that the F116H mutation had little effect on iron uptake. All three tES(+)F116H/tES-POI had greatly reduced (~ 1–2%) iron uptake when compared with wild-type AfFtn and empty tES variants (Supplementary Fig. 7b).

### POI stabilization with tES and release with pH titration

**Fig. 3:**
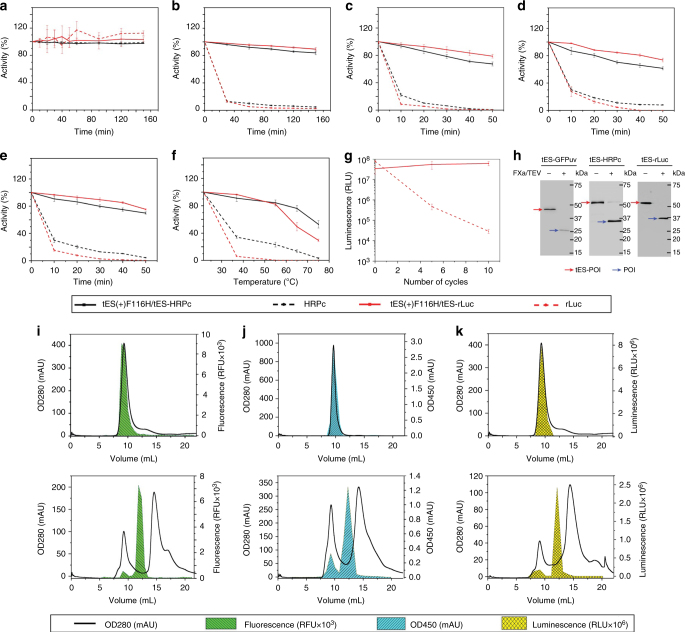
Effect of tES on the stability of the encapsulated POI and the release of the POI with mild pH titration. **a** POI is stable in the dialysis buffer with and without encapsulation. The presence of tES enhanced the stability of tES-POI in **b** 0.4% trypsin, **c** 20% methanol, **d** 8 M urea, and **e** 30% acetonitrile. **f** tES(+)F116H/tES-POI is resistant to 15 min of thermal denaturation. **g** tES(+)F116H/tES-rLuc is highly resistant to repeated thermocycling (80 °C × 5 min × 10 cycles) showing a three order-of-magnitude higher activity compared with rLuc. **h** Recovery of active proteins from the tES. tES(+)F116H/tES-GFPuv, tES(+)F116H/tES-HRPc, and tES(+)F116H/tES-rLuc were subjected to pH 5.8 which resulted in the cage break and release of tES-POI. The released tES-POI (MW of tES-GFP [46 kDa], tES-HRPc [53 kDa], and tES-rLuc [55 kDa]) was proteolyzed by FXa/TEV to separate GFPuv (27 kDa), HRPc (34 kDa), and rLuc (36 kDa) from the tES subunit (19 kDa). The separated POIs were detected on the western blot using c-Myc antibody targeted to the c-Myc epitope downstream of POIs. **i**–**k** Size-exclusion chromatography of tES(+)F116H/tES-GFPuv, tES(+)F116H/tES-HRPc, and tES(+)F116H/tES-rLuc at pH values of 8.0 (upper panels) and 5.8 (lower panels). Each fraction was analyzed for GFPuv, HRPc, and rLuc activity and the data were overlaid on the respective chromatogram. All experiments were performed in triplicates and error bars represent means ± standard deviation (SD)

We tested the ability of tES to impart thermostable qualities to internalized proteins. GFPuv is highly stable in its native form. We therefore tested rLuc and HRPc versus unencapsulated controls to determine the stabilizing effect of tES encapsulation. For both rLuc and HRPc, tES was protective from 0.4% trypsin, 30% acetonitrile, 20% methanol, 8 M urea, 6 M GuHCl, and thermal denaturation (Fig. 3a–g and Supplementary Fig. 7c).

Finally, we tested the encapsulated, functional proteins for their ability to be released from tES(+)F116H with mild pH titration (pH 5.8–6). All three proteins were easily released and purified under SEC, and POIs could be proteolyzed from the fusion tES subunit to produce a monomeric, soluble protein (Fig. 3h–k).

## Discussion

The pathway from a nascent polypeptide to a functional protein structure is determined by a balance of factors, some working in favor of, and many against, the successful folding of the end product^21, 22^. Natural chaperones prevent the aggregation of unfolded or partially folded intermediates, undergo specific interactions that bias folding along productive energetic pathways, and exert kinetic effects that accommodate folding processes of widely varying time scales^23, 24^. In the setting of recombinant expression, the intrinsic chaperone availability in an *E. coli* cell may be overwhelmed by the bulk of a nascent, unfolded recombinant protein^25^. In support of this hypothesis, the overexpression of native chaperones such as Hsp70 and GroEL/GroES complex has aided in recombinant protein expression^26–28^. Chaperone overexpression has also shown limits in its application, as the coexpression of DnaJ, DnaK, and GrpE with HRP inhibited the growth of host cells^29^, and DnaK-assisted expression of GFP resulted in reduced yield and lower conformational quality of the target protein^30, 31^.

Studies using the P22 VLP bacteriophage have shown sequestration of a recombinant protein within the interior surface^32^. However, the requirement of ~ 450 coat proteins, additional scaffold proteins, and the harsh conditions required to disassociate the capsid have limited the use of P22 in recombinant expression^33^.

By using 23 copies of a single thermostable subunit to form a protective shell around internalized proteins, we report that tES can improve expression, in vitro folding, and product stabilization. tES(+)F116H can release the encapsulated protein with mild pH titration (pH 5.8–6.0) and the soluble protein fusions can be selectively proteolyzed to create monomeric protein products. A caveat of this study is that we use protein function as a surrogate for folding. Thus, to understand the precise effects of nanoenvironmental engineering on the folding of protein substrates, further studies such as differential scanning calorimetry, deuterium exchange, and cryoelectron microscopy may be needed. Because each POI substrate is physically isolated from other unfolded proteins via the tES, we hypothesize that folding studies can be performed at a higher concentration without aggregation.

Although ~ 80% of translated proteins in the eukaryotic genome are 80 kDa or smaller^34^, the uses of tES may be broadened by variants with larger internal volumes. This will be particularly important for POIs that require multimerization for optimum activity. The ability to stabilize thermolabile substrates within tES may be helpful for a variety of applications in bionanotechnology and synthetic biology, including the production of difficult-to-fold proteins, using the shell as a mediator of cellular enzyme uptake, and exploiting the stabilized qualities of tES substrates in industrial settings.

## Methods

### Plasmids and competent cells

The pRSF1b expression vector (Merck) and pBAD/HisB vector (Life Technologies) were used for cloning. Chemically competent XL1 Blue *E. coli* cells (catalog #RH119-80; Simply Science) and BL21 (DE3) *E. coli* cells (catalog #RH217-J40; Simply Science) were used for transformation.

### Reagents

The following reagents were used: restriction enzymes *Nco*I, *Eag*I, and *Spe*I (New England BioLabs), Expresslink T4 DNA ligase (Life Technologies), Luria-Bertani (LB) agar (Axil Scientific), kanamycin (ThermoFisher), ampicillin (Axil Scientific), Omega bio-tek plasmid mini kit and gel extraction kit (Simply Science), Q5® Site-Directed Mutagenesis Kit (New England BioLabs), IPTG (Axil Scientific), L-arabinose (Sigma), Tris-HCl at a pH of 8.0 (Sigma), sodium chloride (NaCl, Sigma), Triton-X 100 (Sigma), calcium chloride (CaCl_2_, Sigma), hemin (Sigma), β-mercaptoethanol (Sigma), imidazole (Sigma), L-glutathione oxidized (Sigma), nitric acid (Merck), bathophenanthroline disulfonic acid (Sigma), dithionite (Sigma), phosphate-buffered saline (PBS, Sigma), ferrous ammonium sulfate (Sigma), potassium iodide (Sigma), sodium thiosulfate (Sigma), Tween-20 (ThermoFisher), chemiluminescent HRP substrate (Millipore), 3,3′,5,5′-tetramethylbenzidine (TMB) substrate (ThermoFisher), coelentrazine substrate (Promega), sulfuric acid (H_2_SO_4_, Sigma), magnesium chloride (MgCl_2_, Sigma), ethylenediaminetetraacetic acid (EDTA, Sigma), bovine FXa (Axil Scientific), TEV protease (Sigma), urea (1st Base), GuHCl (Sigma), dithiothreitol (DTT, Sigma), glycerol (Sigma), 0.5% trypsin-EDTA (Life Technologies), methanol (MeOH, Fisher Scientific), acetonitrile (ACN, Fisher Scientific), and Thermo ScientificTM PierceTM c-Myc Tag IP/Co-IP Kit (cat. no. 23620). The following antibodies were used: c-Myc (9E10) HRP mouse monoclonal IgG1 (catalog #sc-40 HRP, Santa Cruz Biotechnology), His-probe (H-3) HRP mouse monoclonal IgG1 (catalog #sc-8036, Santa Cruz Biotechnology), rabbit monoclonal GFP antibody (catalog #G10362; Invitrogen), and HRP-conjugated anti-rabbit IgG antibody (catalog #A10260; Invitrogen).

### Cloning

AfFtn gene was selected based on the sequence in GenBank AF_RS04235. The gene with mutations for C-terminus truncation, as well as altered charges was synthesized from GenScript. The mutated gene was digested using *Nco*I and *Spe*I restriction enzymes and cloned into pRSF1b expression vector using Expresslink T4 DNA ligase. The ligation reaction was transformed into chemically competent XL1 Blue *E. coli* cells and cultured on LB agar plates with 25 µg/mL kanamycin. Plasmid DNA was isolated by Omega bio-tek plasmid mini kit and gel extraction kit, and the sequence was confirmed by nucleic acid sequencing. The engineered AfFtn genes were used as the template for F116H mutations using Q5® Site-Directed Mutagenesis Kit with the following primers: F116H forward 5′-GCGATGCAGGAAAAAGATCATGCGACCTATAACTTTCTG-3′ and F116H reverse 5′-TATGCTGGCGGGCCCGCAGATGCATGGTACTAGTTCCATGGTGGTCAAAATCTGCGGCATCAAAAGCCTGGAAGAACTGGAAATCGTGGAAAAACACGCGGATGCCA-3′. The genes for GFPuv (based on the wild-type GFP sequence P42212 with three mutations F99S, M153T, and V163A), HRPc (P00433), and rLuc (P27652) were synthesized from Genscript, digested using *Spe*I and *Eag*I restriction enzymes, and the resulting fragments ligated with pBAD/HisB using Expresslink T4 DNA ligase. The gene for engineered AfFtn was ligated into the pBAD/HisB containing the gene for POI. The ligation reaction was transformed into chemically competent XL1Blue *E. coli* cells. The plasmid DNA was isolated and sequenced. The pRSF1b (with a T7 promoter plasmid and a complementary RSF origin of replication) and pBAD/HisB (with an L-arabinose promoter and a pBR322 origin of replication) constructs were transformed into BL21(DE3) *E. coli* cells and grown on LB agar plates with 25 µg/mL kanamycin and 50 µg/mL ampicillin, respectively. For in vivo studies, pRSF1b and pBAD/HisB constructs were cotransformed in BL21(DE3) *E. coli* cells and grown in a medium containing 50 µg/mL kanamycin and 100 µg/mL ampicillin.

### Protein expression in *E. coli*

For the expression of each clone, a single positive colony was selected from a freshly transformed plate and grown in 100-mL LB broth, with kanamycin (50 µg/mL, for a protein gene on pRSF1b vector), ampicillin (100 µg/mL, for a protein gene on pBAD/HisB vector), or both (cotransformation) used as selection markers. Following overnight incubation at 37 °C, 12.5 mL of the starter culture was used to inoculate a 500-mL LB broth and allowed to grow until an absorbance (OD600) of 0.4–0.5 was reached. Protein expression was then induced with 0.4 mM IPTG (pRSF vector), 0.1% L-arabinose (pBAD vector), or both (cotransformation). We tested the role of a tightly controlled relative expression of the encapsulated POI by evaluating the effect of L-arabinose (0.01, 0.1, and 1%) on the functional expression of GFPuv. After 4 h of incubation at 37 °C, cells were pelleted by centrifugation at 13,750 ×**g** for 10 min. The cell pellet was resuspended in a lysis buffer (25 mM Tris-HCl, 150 mM NaCl, pH 7.5), sonicated, and centrifuged to separate cell debris. The supernatant obtained was purified using a two-step chromatography procedure. pRSF clones were subjected to hydrophobic interaction chromatography (HIC) using HiPrepTM Phenyl FF (low sub) 16/10 (GE healthcare), followed by SEC using a Superdex S-200 10/300 GL column (GE healthcare). pBAD clones were subjected to Ni-NTA (Expedeon) column chromatography, followed by SEC. Fusion proteins were purified using Ni-NTA and SEC. The purity of the SEC fraction was analyzed using sodium dodecyl sulfate polyacrylamide gel electrophoresis (SDS-PAGE). Buffers used were as follows: HIC: buffer A, 25 mM Tris-HCl, 150 mM NaCl, and 1 M (NH_4_)_2_SO_4_, pH 7.5; buffer B, 25 mM Tris-HCl, 150 mM NaCl, pH 7.5; Ni-NTA chromatography: buffer A, 25 mM Tris-HCl, 150 mM NaCl, pH 7.5; buffer B, 25 mM Tris-HCl, 150 mM NaCl, and 500 mM imidazole, pH 7.5; and SEC: 25 mM Tris-HCl, pH 8. All buffers used for the lysis, purification, and assays of HRPc clones were supplemented with 5 mM CaCl_2_ and 2.5 µM hemin (a stock solution of 40 mM was prepared by dissolving 25 mg of hemin in 1 mL of 1.4 N ammonium hydroxide).

### Protein concentration and iron assay

Protein concentration was determined using Beer–Lambert’s equation by measuring the absorbance at 280 nm using Nanodrop (DeNovix) and a molar extinction coefficient. The total iron content in the engineered tES was determined spectrophotometrically^9^. Protein samples were denatured by treating with 50 mM nitric acid and mixed with 10 mM bathophenanthroline disulfonic acid, 20 mM dithionite, and 250 mM Tris buffer, at a pH of 8.0. The mixture was incubated overnight and iron concentration was measured from the absorbance of the complex at 538 nm (ϵ_538_ = 22.1 mM^−1^∙cm^−1^). For iron uptake study, an equimolar concentration of samples (1 µM) was treated with ferrous ammonium sulfate (100 mM), potassium iodide (50 mM), and sodium thiosulfate (200 mM). The reaction was incubated at room temperature for 20 min, followed by dialysis with 1% ammonium sulfate. The iron concentration was measured from the absorbance of the solution at 538-nm wavelength^20^.

### Size determination

Particle size measurements of engineered NEs and wild-type AfFtn were conducted by dynamic light scattering (DLS) technique using a zetasizer (Nano-ZS90, Malvern) in disposable cuvettes, and the average hydrodynamic diameter was determined by taking an arithmetic average of 10 runs. Measurements were performed for 100 µM protein at 25 °C.

### Western blot analysis

Samples (purified protein or cell lysate) were resolved on a 12% SDS gel and transferred onto a nitrocellulose membrane using i-blot apparatus (Life Technologies). The membrane was washed with 1× PBS, dried, and blocked overnight with 5% blotting-grade blocker in PBST (PBS with 0.05% Tween-20). The block was removed and the membrane was incubated with antibodies (c-Myc [9E10] HRP, mouse monoclonal IgG1, His-probe [H-3] HRP, or mouse monoclonal IgG1, each at 1:1000 dilution). After 30-min incubation on a rocker, the membrane was washed thrice with PBST and protein bands were detected with a chemiluminescent HRP substrate.

### Thermal unfolding studies

Far-UV CD spectra (260–190 nm) were recorded using a Chirascan Circular Dichroism Spectrometer (Applied Photophysics). Protein samples (0.1 mg/mL) were dissolved in 10 mM phosphate buffer and measurements were carried out at room temperature using a 0.1-cm path length-stoppered cuvette. The heat-induced denaturation of proteins was conducted by heating protein solutions at the rate of 1 °C/min and the spectra were recorded for every 1 °C change.

### Cage break of the pH-responsive tES(+)F116H shells

Following the purification of the tES(+)F116H shells, the protein was acidified for shell disassembly in 25 mM Tris-citrate buffer, at a pH of 5.8 for 30 min. The sample was subjected to SEC using 25 mM Tris-citrate buffer at a pH of 5.8 and the disassembled tES subunits were collected for in vitro folding.

### Inclusion body POI expression and solubilization

Following the expression of each POI, cells were pelleted by centrifugation at 13,750 ×g for 10 min. The cell pellet was resuspended in a lysis buffer (1.5% Triton X-100, 25 mM Tris-HCl, and 150 mM NaCl, pH 7.5–8), sonicated, and centrifuged to separate the cell pellet. The cell pellet was resuspended in washing buffers (0.5% Triton X-100, 25 mM Tris-HCl, and 200 mM NaCl, pH 8 and 25 mM Tris-HCl, 0.5 M NaCl, and 2 M urea, pH 8) and centrifuged to separate the cell pellet. The pellet was resuspended in a solubilization buffer (25 mM Tris-HCl, 6 M GuHCl, and 0.5 M NaCl, pH 8) to solubilize inclusion bodies, followed by centrifugation at 13,750 ×g for 20 min for the separation of cell debris. The supernatant obtained was purified using Ni^2+^-NTA column equilibrated with buffer A. The bound protein was eluted with buffer B. The purity of the eluted fraction was analyzed using SDS-PAGE.

### In vitro folding

The POI sample (1 μM) was heated at 60 °C for 30 min, followed by its pH adjustment to 5.8 using 6 M GuHCl. The POI was incubated in the presence of tES(+) subunits (in 25 mM Tris-HCl), with the subunit-to-POI ratio of 90:1, 60:1, 30:1, 20:1, 10:1, and 0:1. The pH of the mixture was adjusted to 8 and the sample was incubated at room temperature for 30 min. The mixture was dialyzed in a refolding buffer (25 mM Tris-HCl, 150 mM NaCl, pH 8; HRPc: 25 mM Tris-HCl, 0.6 M GuHCl, 0.35 mM oxidized glutathione, 0.044 mM DTT, 7% glycerol, 5 mM CaCl_2_, and 20 μM heme, pH 8.5) using Slide-A-Lyzer® Dialysis Cassette G2 (Thermo Scientific). The refolded protein was purified using Ni-NTA chromatography, followed by SEC as described above. Fractions around the eluted peak were collected and their activity was analyzed to identify fractions containing the POI.

### In vitro assay

GFPuv, HRPc, and rLuc activities were determined through fluorescence, colorimetric, and luminescence assays, respectively, from purified proteins. All reactions were performed at least in triplicates. For GFPuv, fluorescence of tES(+)F116H/tES-GFPuv, tES-GFPuv, and GFPuv was read at 508 nm in a 96-well black polystyrene plate (Fisher Scientific) with an excitation wavelength fixed at 395-nm wavelength. The activity of tES(+)F116H/tES-HRPc, tES-HRPc, and HRPc was assayed using TMB substrate in a 96-well crystal-clear polystyrene plate (Greiner Bio-One). Purified fractions were incubated in an assay buffer containing 25 mM Tris-HCl at a pH of 8.0, 5 mM CaCl_2_, and 2.5 µM hemin for 5 min. The TMB substrate was added for color development and the reaction stopped using 2 M H_2_SO_4_ after 5 min. Absorbance was recorded at 450 nm. All luciferase reactions took place at ambient temperature (24–27 °C) in a 96-well plate with a white interior. Following chromatographic purification, equimolar concentrations (500 nM) of purified tES(+)F116H/tES-rLuc, tES-rLuc, and rLuc were evaluated for luciferase activity using *Renilla* luciferase kit (Promega) with some modifications in the manufacturer’s instructions. The reaction was initiated by injecting 50 µL of *Renilla* luciferase assay reagent (1:1,000 dilution of coelentrazine in the assay buffer). The assay reagent was protected from light at all times by covering the tubes with an aluminum foil. The signal was integrated for 1 min with a 2-s delay and was reported in RLU. All readings were recorded on a Perkin Elmer Plate reader. Appropriate controls were used in each case to minimize the background.

### Release of a functional protein

Engineered pH-responsive tES(+)F116H shells containing the POI—GFPuv, HRPc, and rLuc—were subjected to cage break as described above. Following cage break, GFPuv, HRPc, and rLuc activity of each fraction was analyzed, as described earlier. The release of the functional POI from the tES subunit was studied by cleavage with bovine FXa/TEV protease. Briefly, the cage break fraction corresponding to the elution of tES-POI on SEC was subjected to FXa cleavage at 37 °C for 4 h (TEV cleavage at 34 °C for 5 h). The reaction mixture was run on an SDS gel and the separated POI band was analyzed through western blot.

### Thermostability and heat shock tests

Concentrations of 0.5 μM tES(+)F116H/tES-HRPc or tES(+)F116H/tES-rLuc, 50 μM HRPc, and 80 μM rLuc protein samples were incubated in an assay buffer (25 mM Tris-HCl, pH 8.0) at 21.5, 37, 55, 65, and 75 °C for 15 min. Following incubation, samples were cooled down and their activities were evaluated. For a heat shock test, 0.5 μM tES(+)F116H/tES-rLuc and 80 μM rLuc protein samples were incubated in 25 mM Tris-HCl (pH 8) at 80 °C for 5 min, followed by cooling the protein samples at 0 °C for 5 min. The process was repeated for 5 and 10 cycles, followed by evaluation of protein activity.

### Trypsin digestion

Concentrations of 0.5 μM tES(+)F116H/tES-HRPc or tES(+)F116H/tES-rLuc, 50 μM HRPc, and 80 μM rLuc protein samples were treated with 0.4% trypsin-EDTA solution at 37 °C for 0, 30, 60, 90, 120, and 150 min, followed by analysis of their activities.

### Analysis of protein stability

The effect of urea, GuHCl, ACN, and MeOH on protein stability was evaluated by treating 0.5 μM tES(+)F116H/tES-HRPc or tES(+)F116H/tES-rLuc, 50 μM HRPc, and 80 μM rLuc protein samples with an assay buffer (pH 8) containing 8 M urea, 6 M GuHCl, 30% ACN, and 20% MeOH, respectively, at 21.5 °C (45 °C for MeOH) for 0, 10, 20, 30, 40, and 50 min. After incubation, the protein samples were buffer exchanged with the assay buffer and their activities were assessed.

### Data availability

All relevant data are available from the authors upon request.

## Electronic supplementary material

Supplementary Information
