# Supplementary material for: Thermostable exoshells fold and stabilize recombinant proteins
Source: Nat Commun. 2017 Nov 13;8:1442. doi: 10.1038/s41467-017-01585-2 (PMC5682286; doi:10.1038/s41467-017-01585-2)
Supplement: Supplementary file 1 — Supplementary Information [file 41467_2017_1585_MOESM1_ESM.pdf]

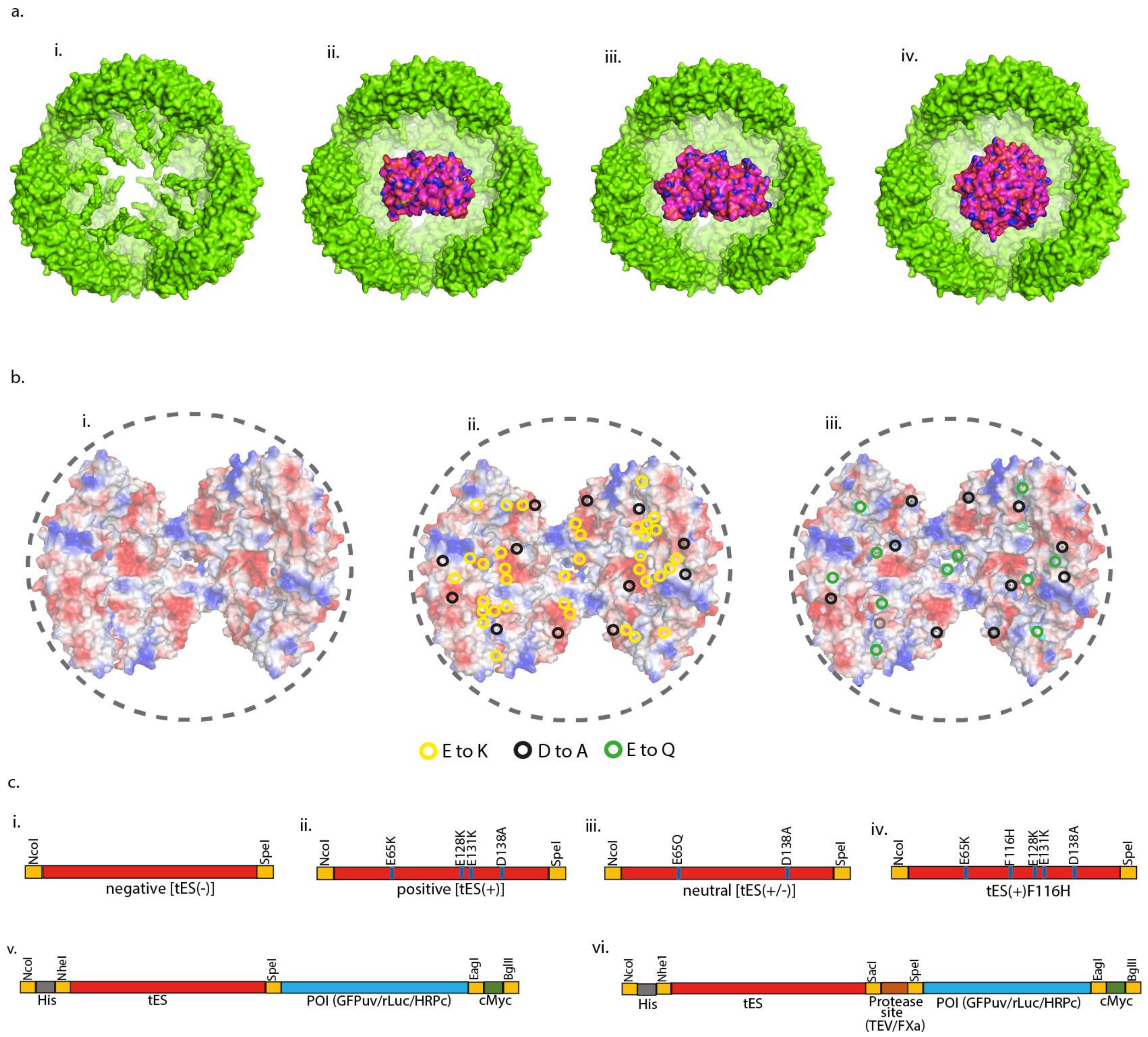

Supplementary Figure 1
Encapsulation of proteins of interest (POI) inside the thermostable exoshell.
(**a**) Surface representations of (i) the 24-subunit wild-type AfFtn assembly (1SQ3) with six subunits digitally removed and the unstructured *C*-termini added as a space filling surface. Surface representations of the 24-subunit engineered tES assembly with six subunits digitally removed and a rendered surface of (ii) green fluorescent protein (27 kDa, 2YOG), (iii) horseradish peroxidase isoenzyme C (34 kDa, 2ATJ), and (iv) *Renilla* luciferin 2-monooxygenase (36 kDa, 2PSD) inside the volume of the shell. (**b**) Molecular surface rendering of 12 subunits of AfFtn with truncated *C*-termini (tES) (PDB accession code 1SQ3). (i) Negative [tES(-)] with negative interior surface. (ii) Positive [tES(+)] with a positive interior surface; a total of 48 mutations (four per subunit: E65K, E128K, E131K, and D138A) are highlighted. (iii) Neutral [tES(+/-)] with a neutral interior surface; a total of 24 mutations (two per subunit: E65Q

and D138A) are highlighted. (**c**) Schematic representation of representative constructs with restriction sites. (i) Negative [tES(-)], AfFtn with truncated *C*-terminus contributing to a net negative interior when assembled. (ii) Positive [tES(+)], AfFtn with truncated *C*-terminus and four point mutations (E65K, E128K, E131K, and D138A) contributing to a net positive interior when assembled. (iii) Neutral [tES(+/-)], AfFtn with truncated *C*-terminus and two point mutations (E65Q and D138A) contributing to a net neutral interior when assembled. (iv) tES(+)F116H, AfFtn with truncated *C*-terminus, a positive interior, and an F116H mutation for studying the effect of pH on shell disassembly. Constructs (i), (ii), (iii), and (iv) were cloned in a pRSF1b vector. (v) tES-POI, fusion of tES subunit and proteins of interest (POI; GFPuv/HRPc/rLuc). *N*-terminus histidine tag and *C*-terminus c-Myc epitope are highlighted. (vi) tES-protease-POI, fusion of tES subunit and POI having a protease site (TEV or FXa) in between. Both constructs (v) and (vi) were cloned in pBAD/HisB vector.

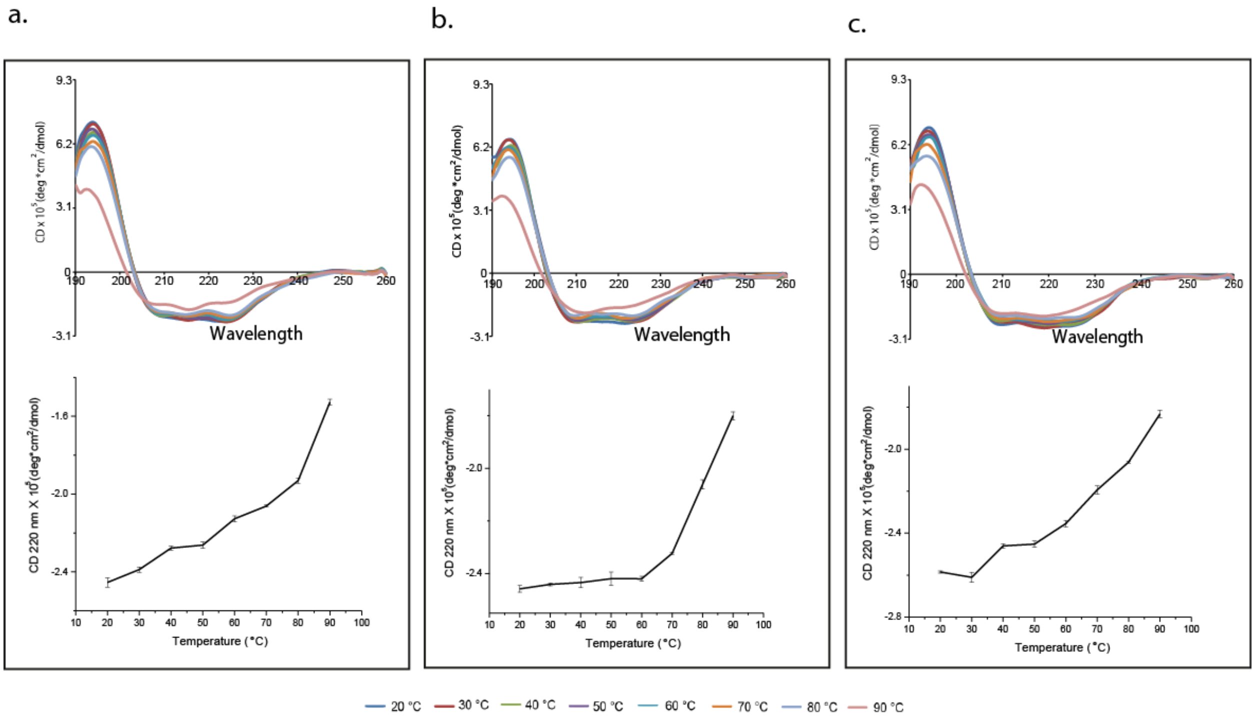

Supplementary Figure 2
Thermal stability of the three engineered exoshells.
Thermal unfolding studies with tES from 20°C to 90°C. (**a**) tES(+), (**b**) tES(-), (**c**) tES(+/-). Far-UV CD spectrum of the shells showing intense minima at 208 and 224 nm and maximum at 195 nm typical of α-helical structure. The protein sample (0.1 mg/mL) was dissolved in 10 mM phosphate buffer and the measurement recorded with a Chirascan Circular Dichroism Spectrometer using a 0.1-cm path length stoppered cuvette. The heat-induced denaturation of proteins was conducted by heating protein solutions at the rate of 1°C/min and the spectra collected for every 1°C change. The lower panel shows the plot of molar ellipticity at 220 nm versus temperature. All experiments were performed in triplicates, error bar represents ± standard deviation.

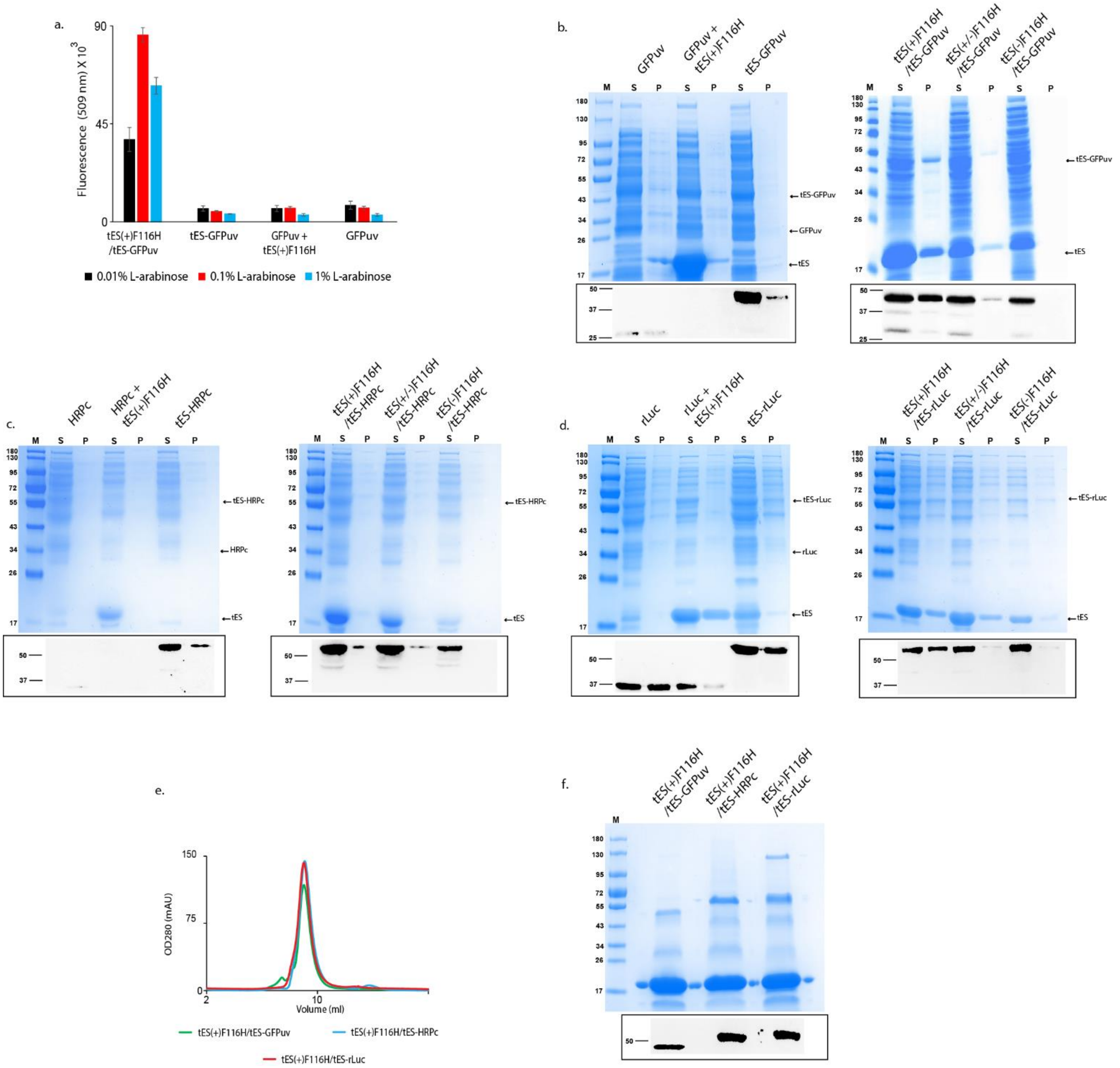

Supplementary Figure 3

Soluble expression and purification of proteins of interest (POI) in presence of exoshells.

(**a**) Comparison of fluorescence at 509 nm for GFPuv clones expressed at three different L-arabinose concentrations. (**b**) SDS-PAGE gel and corresponding western blot of GFPuv expressed as a His-tagged protein, His-tagged GFPuv without being fused to a tES subunit (linker) co-expressed with tES shell, GFPuv fused to the tES subunit expressed without tES shell, His-tagged GFPuv fused to the tES subunit and co-expressed with positive, neutral, and negative tES shell. (**c**) SDS-PAGE gel and corresponding western blot of HRPc expressed as a His-tagged protein, His-tagged HRPc without being fused to a tES subunit (linker) co-expressed

with tES shell, HRPc fused to the tES subunit expressed without tES shell, His-tagged HRPc fused to the tES subunit and co-expressed with positive, neutral, and negative tES shell. (**d**) SDS-PAGE gel and corresponding western blot of rLuc expressed as a His-tagged protein, His-tagged rLuc without being fused to a tES subunit (linker) co-expressed with tES shell, rLuc fused to the tES subunit expressed without tES shell, His-tagged rLuc fused to the tES subunit and co-expressed with positive, neutral, and negative tES shell. (**e**) Size-exclusion profiles of purified complexes (1 μM), wherein the POI are GFPuv, HRPc, and rLuc. In all cases, the His-tagged subunit co-purifies with tES shell. (**f**) SDS-PAGE gel and the corresponding western blot of the purified GFPuv, HRPc, and rLuc showing bands of tES-POI and tES subunit. All experiments were performed in quadruplicates, error bar represents ± standard deviation.

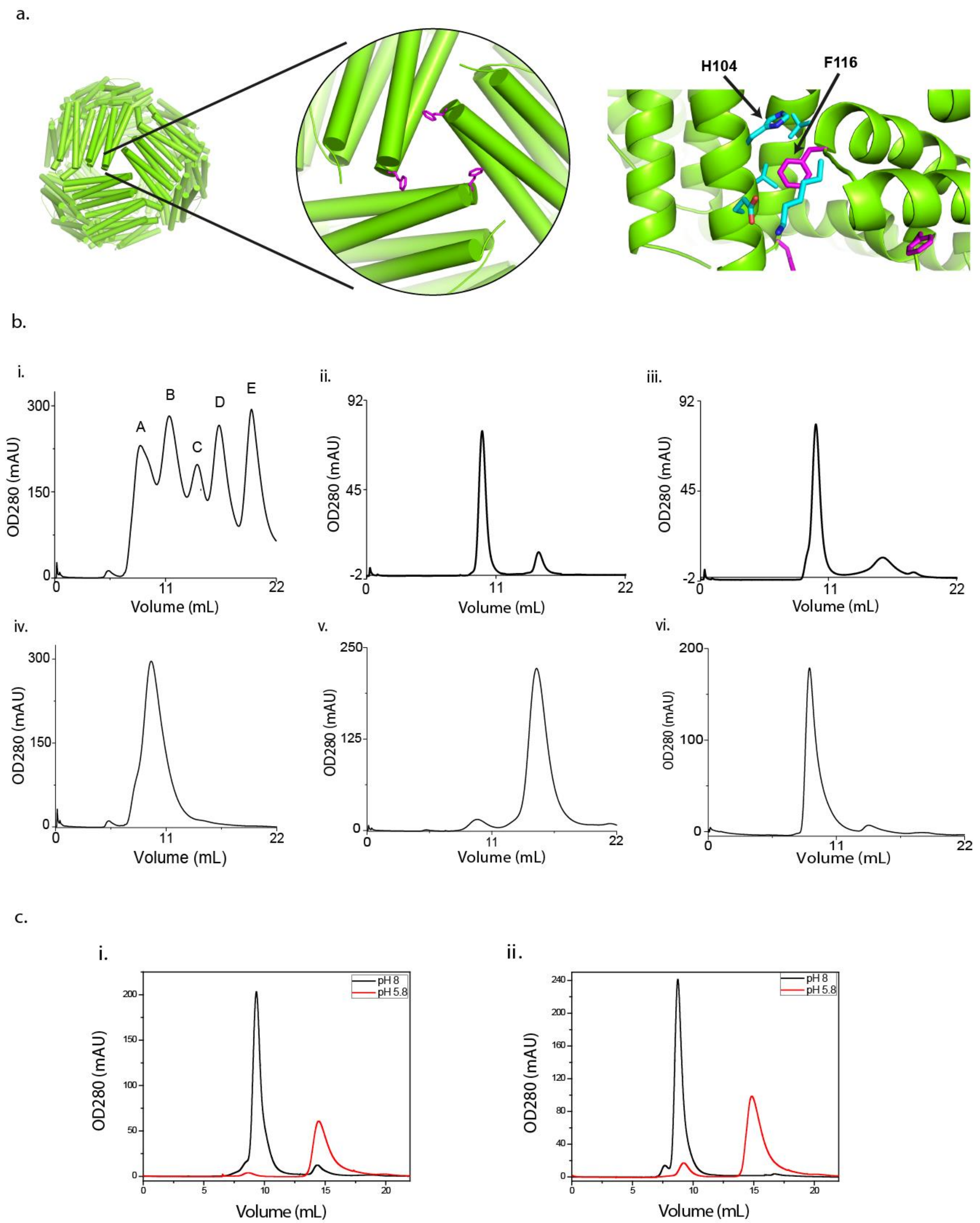

Supplementary Figure 4

Effect of F116H mutation on shell assembly.

(**a**) F116 along the three-fold symmetry axis and its substitution to histidine. The helices are represented as green cylinders. (**b**) Effect of F116H mutation on shell disassembly. All size

exclusion chromatography experiments were performed with 25 mM Tris-citrate pH 8.0 and 5.8 buffers. (i) Chromatogram for size-exclusion standard components (A= thyroglobulin, MW 670 kDa; B= γ-globulin, MW 158 KDa; C= ovalbumin, MW 44kDa; D= myoglobin, MW 17 kDa; E= Vitamin B_{12}, MW 1.3 KDa). (ii) and (iii) Chromatograms of tES(+) at pH 8.0 and pH 5.8, respectively. (iv) and (v) Chromatograms of tES(+)F116H at pH 8.0 and pH 5.8, respectively. At pH 5.8, the tES(+)F116H disassembles into its subunits as indicated by peak 2. (vi) The pH of the subunit sample was increased to 8 using 25 mM Tris-citrate buffer, followed by size-exclusion chromatographic (SEC) separation to show reversible assembly of the cage. (**c**) SEC separation of tES(+)F116H at pH 8.0 and 5.8 in presence of 6 M guanidinium HCl (i) and 8 M urea (ii). High concentration of denaturants showed no effect on tES(+)F116H disassembly at pH 5.8. All experiments were performed in triplicates and error bar represents ± standard deviation.

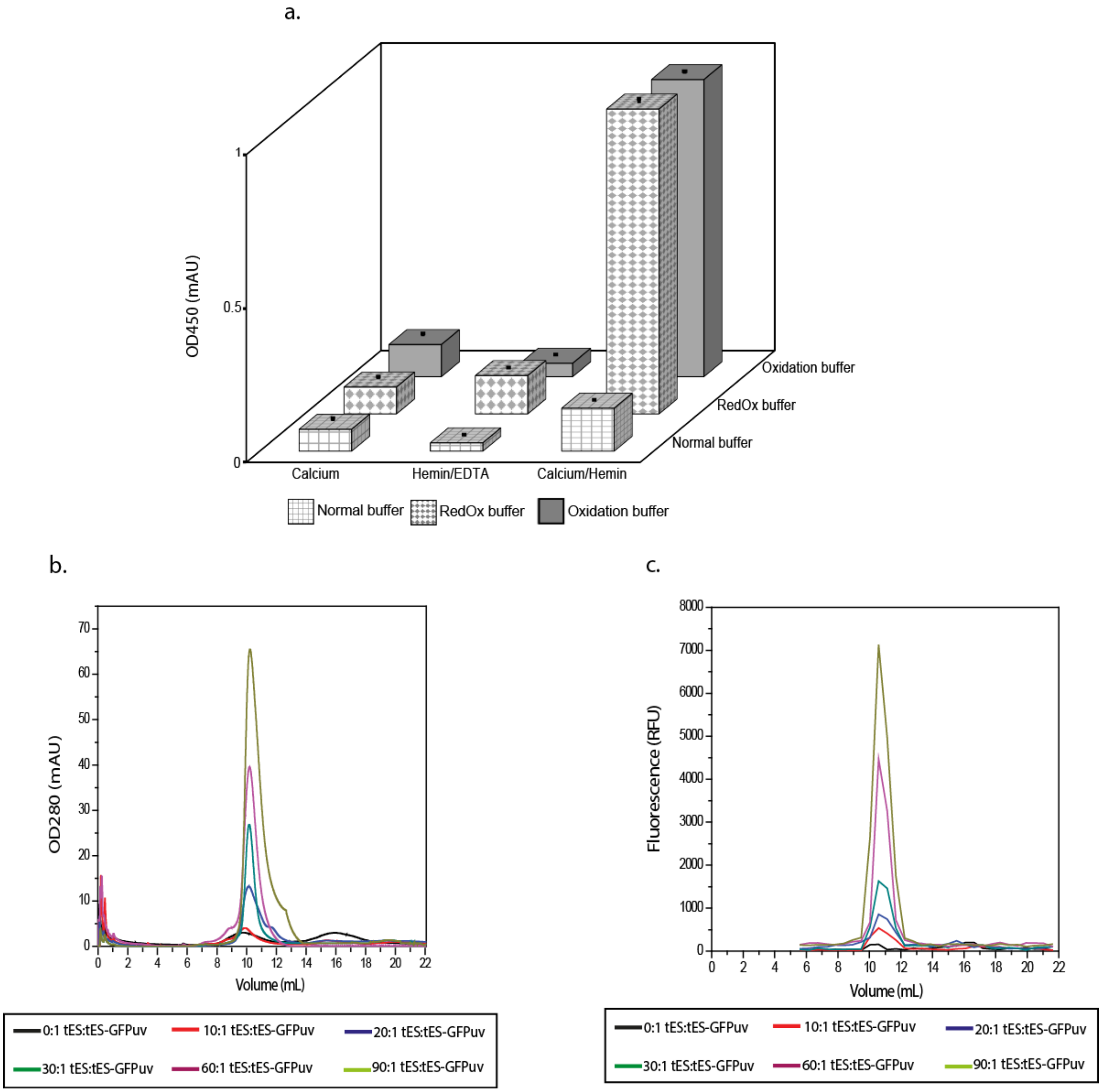

Supplementary Figure 5

Effects of cofactors/additives on HRPc activity and different concentrations of tES subunits on GFP folding.

(**a**) Effect of cofactors/additives on HRPc activity inside the tES. HRPc activity was assessed from cell lysates processed under identical conditions in presence of cofactors calcium and hemin in addition to oxidized and reduced glutathione additives (n=3, error bar represents ± SD). Normal buffer, purification in a buffer with no reducing/oxidizing agent; RedOx buffer, purification in a reducing buffer with 10 mM β-mercaptoethanol, followed by buffer exchange with an oxidation buffer containing 0.3 mM oxidized glutathione; Oxidation buffer, purification directly in oxidation buffer. (**b**) Size-exclusion profile of tES(+)F116H/tES-GFPuv with different

molar ratios of tES subunits and tES-GFPuv. In all cases, the histidine-tagged subunit co-purifies with tES, indicating its encapsulation inside the tES assembly. (**c**) Each fraction of the size exclusion chromatography was analyzed for its GFPuv activity for all molar ratios tested. All experiments were performed in triplicates, error bar represents ± standard deviation.

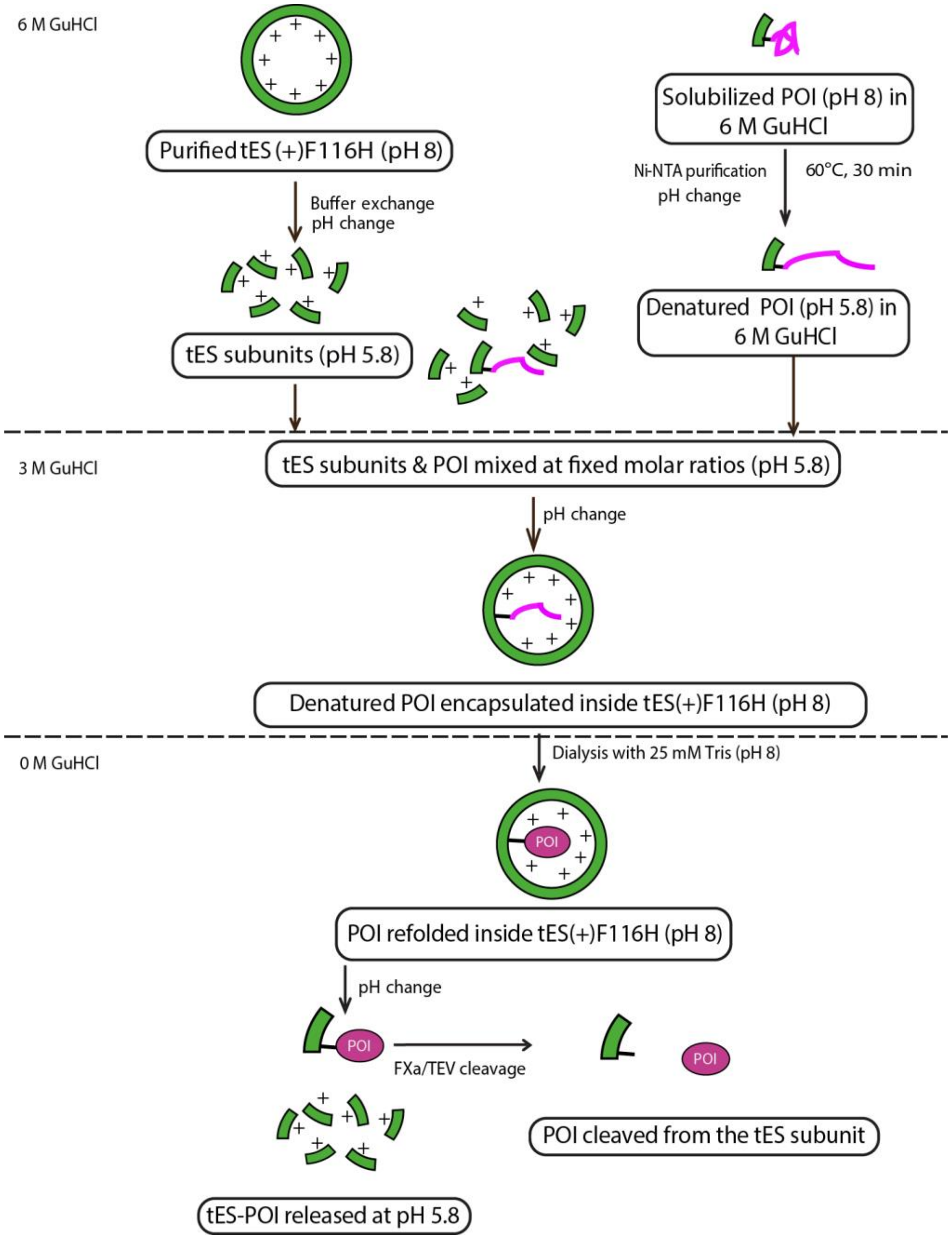

Supplementary Figure 6
Protocol for in vitro folding of proteins of interest (POI).
The tES-POI was solubilized in 6 M GuHCl and heated at 60°C to ensure its complete denaturation. Following denaturation, tES-POI mixed with tES subunits at fixed molar ratio at pH 5.8. The pH was gradually changed to 8 to ensure encapsulation of the denatured protein inside tES(+)F116H. The POI was subjected to dialysis using 25 mM Tris buffer, pH 8, for its folding inside tES(+)F116H.

a.

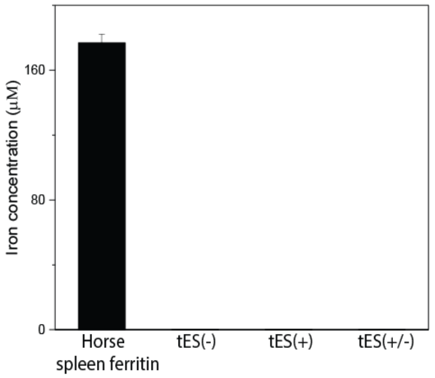

b.

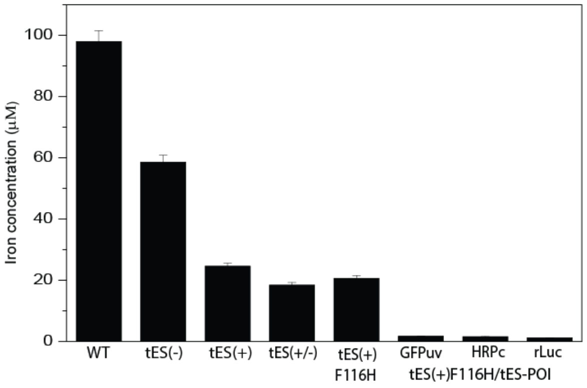

	Iron concentration (μM)
Horse spleen ferritin	176.98 ± 5.32
tES(-)	0.40 ± 0.019
tES(+)	0.39 ± 0.038
tES(+/-)	0.26 ± 0.034

	Iron concentration (μM)
WT	97.97 ± 3.51
tES(-)	58.61 ± 2.21
tES(+)	24.65 ± 0.90
tES(+/-)	18.45 ± 0.85
tES(+)F116H	20.66 ± 0.88
tES(+)F116H/tES-GFPuv	1.73 ± 0.09
tES(+)F116H/tES-HRPc	1.61 ± 0.02
tES(+)F116H/tES-rLuc	1.20 ± 0.03

c.

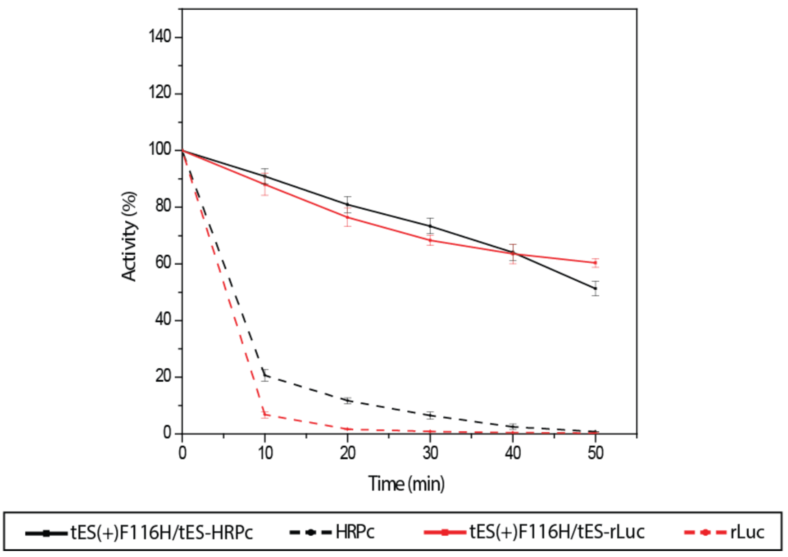

Supplementary Figure 7

Iron uptake study and effects of guanidinium hydrochloride on POI stability.

(**a**) Iron assay - Iron concentration of the three engineered exoshells (1 μM concentration) was assayed with horse spleen ferritin as a positive control. The iron content in the engineered exoshells was negligible as compared with that in the positive control. (**b**) Iron uptake study of ferritin shells using ferrous ammonium sulfate. In comparison to the wild type Afu ferritin, the

engineered tES shells showed lower iron uptake. All experiments were performed in triplicates, error bar represents ± standard deviation. (**c**) tES protects HRPc and rLuc from strong denaturant, 6 M GuHCl. All experiments were performed in triplicates, error bar represents ± standard deviation.
